# Effect of Roux-en-Y Bariatric Surgery on Lipoproteins, Insulin Resistance, and Systemic and Vascular Inflammation in Obesity and Diabetes

**DOI:** 10.3389/fimmu.2017.01512

**Published:** 2017-11-15

**Authors:** Rahul Yadav, Salam Hama, Yifen Liu, Tarza Siahmansur, Jonathan Schofield, Akheel A. Syed, Michael France, Philip Pemberton, Safwaan Adam, Jan Hoong Ho, Reza Aghamohammadzadeh, Shaishav Dhage, Rachelle Donn, Rayaz A. Malik, John P. New, Maria Jeziorska, Paul Durrington, Basil A. Ammori, Handrean Soran

**Affiliations:** ^1^Cardiovascular Research Group, Core Technologies Facility, The University of Manchester, Manchester, United Kingdom; ^2^Department of Metabolism, Endocrinology and Diabetes, Central Manchester University Hospitals NHS Foundation Trust, Manchester, United Kingdom; ^3^Department of Endocrinology and Diabetes, Salford Royal NHS Foundation Trust, Salford, United Kingdom; ^4^Faculty of Biology, Medicine and Health, The University of Manchester, Manchester, United Kingdom; ^5^Department of Biochemistry, Central Manchester University Hospitals NHS Foundation Trust, Manchester, United Kingdom; ^6^The Division of Musculoskeletal and Dermatological Sciences, The University of Manchester, Manchester, United Kingdom; ^7^Weill Cornell Medicine-Qatar, Doha, Qatar; ^8^Department of Surgery, Salford Royal NHS Foundation Trust, Salford, United Kingdom

**Keywords:** Roux-en-Y, lipoproteins, insulin resistance, vascular inflammation, diabetes

## Abstract

**Purpose:**

Obesity is a major modifiable risk factor for cardiovascular disease. Bariatric surgery is considered to be the most effective treatment option for weight reduction in obese patients with and without type 2 diabetes (T2DM).

**Objective:**

To evaluate changes in lipoproteins, insulin resistance, mediators of systemic and vascular inflammation, and endothelial dysfunction following Roux-en-Y bariatric surgery in obese patients with and without diabetes.

**Materials and methods:**

Lipoproteins, insulin resistance, mediators of systemic and vascular inflammation, and endothelial dysfunction were measured in 37 obese patients with (*n* = 17) and without (*n* = 20) T2DM, before and 6 and 12 months after Roux-en-Y bariatric surgery. Two way between subject ANOVA was carried out to study the interaction between independent variables (*time since surgery* and *presence of diabetes*) and all dependent variables.

**Results:**

There was a significant effect of *time since surgery* on (large effect size) weight, body mass index (BMI), waist circumference, triglycerides (TG), small-dense LDL apolipoprotein B (sdLDL ApoB), HOMA-IR, CRP, MCP-1, ICAM-1, E-selectin, P-selectin, leptin, and adiponectin. BMI and waist circumference had the largest impact of *time since surgery*. The effect of *time since surgery* was noticed mostly in the first 6 months. Absence of diabetes led to a significantly greater reduction in total cholesterol, low-density lipoprotein cholesterol, and non-high-density lipoprotein cholesterol although the effect size was small to medium. There was a greater reduction in TG and HOMA-IR in patients with diabetes with a small effect size. No patients were lost to follow up.

**Conclusion:**

Lipoproteins, insulin resistance, mediators of systemic and vascular inflammation, and endothelial dysfunction improve mostly 6 months after bariatric surgery in obese patients with and without diabetes.

**Clinical Trial Registration:**

www.ClinicalTrials.gov, identifier: NCT02169518. https://clinicaltrials.gov/ct2/show/NCT02169518?term=paraoxonase&cntry1=EU%3AGB&rank=1.

## Introduction

Globally, mean body mass index (BMI) has increased progressively since 1980 (1). Moreover, a 33% increase in obesity prevalence and a 130% increase in severe obesity prevalence have been projected over the next 2 decades (2). Excess mortality above the BMI range of 22.5–25 kg/m^2^ is mainly attributed to vascular disease (3). The outcome of the Swedish obese subjects (SOS) trial indicated that bariatric surgery, when compared with usual care, was associated with a long-term reduction in overall mortality and reduced incidence of type 2 diabetes (T2DM), myocardial infarction, stroke, and cancer (4). Indeed 72% of patients with T2DM at baseline were in remission 2 years after surgery and high baseline insulin and/or high glucose predicted favorable treatment effects, whereas high BMI did not (4).

There is evidence that high-sensitivity C-reactive protein (hsCRP), tumor necrosis factor-alpha (TNFα), monocyte chemoattractant protein 1 (MCP-1), intercellular adhesion molecule 1 (ICAM-1), E-selectin, P-selectin, resistin, and leptin may be mediators of insulin resistance, vascular inflammation, and endothelial dysfunction in those with obesity (5–9). It has been suggested that adiponectin increases insulin sensitivity and reduces the risk of atherosclerosis (10). Leptin and adiponectin are primarily released from the adipocytes whereas hsCRP, TNFα, MCP-1, and ICAM-1 are chiefly released from non-fat cells in adipose tissue (11).

While some studies have shown a significant reduction in mediators of vascular inflammation after bariatric surgery at 6 months (12) and 12 months (13–16), but not at 1 month (17). Other studies have reported no significant change in mediators of vascular inflammation 6 months after bariatric surgery (18).

In this study, we hypothesized a significant improvement in lipids, lipoproteins, insulin resistance, mediators of systemic and vascular inflammation, and endothelial dysfunction at 6 and 12 months after Roux-en-Y bariatric surgery in obese patients with and without diabetes. Outcomes:

1. Effect size of time since surgery on dependent variables associated with lipoproteins, insulin resistance, mediators of systemic and vascular inflammation, and endothelial dysfunction at 6 and 12 months after Roux-en-Y bariatric surgery (as small, medium, and large).
2. Effect size of presence/absence of diabetes on dependent variables associated with lipoproteins, insulin resistance, mediators of systemic and vascular inflammation, and endothelial dysfunction at 6 and 12 months after Roux-en-Y bariatric surgery (as small, medium, and large).

## Materials and Methods

### Participants

The study was approved by the local research ethics committee. All procedures performed in studies involving human participants were in accordance with the ethical standards of the institutional and/or national research committee and with the 1964 Helsinki declaration and its later amendments or comparable ethical standards. We recruited 37 obese patients (17 with T2DM) awaiting bariatric surgery at Salford Royal Hospital (Salford, UK). Informed consent was obtained from all individual participants included in the study. Patients with anemia, acute coronary syndrome within 6 months, any malignancy, active infections, autoimmune diseases, HIV, and other chronic diseases were excluded. All smokers were required to stop smoking at least 2 weeks prior to the date of surgery. Each participant attended The Wellcome Trust Clinical Research Facility (Manchester, UK) where blood samples were taken at baseline, 6, and 12 months. Hypertension was defined as clinic blood pressure 140/90 mmHg or higher and subsequent ambulatory blood pressure monitoring daytime average or home blood pressure monitoring average blood pressure of 135/85 mmHg or higher (NICE Clinical Guideline 127, https://www.nice.org.uk/guidance/CG127/chapter/1-Guidance#diagnosing-hypertension-2).

### Separation of Serum and Plasma

Blood samples were collected between 09:00 and 11:00 hr after participants had fasted from 22:00 hr the previous day. Serum and EDTA-plasma were isolated by centrifugation at 2,000 × *g* for 15 min at 4°C within 2 h of collection and were maintained at that temperature until further use. Aliquots for biochemical analysis were frozen at −80°C.

### Laboratory Analyses

Total cholesterol (TC) was measured using the cholesterol oxidase phenol 4-aminoantipyrine peroxidase method, triglycerides (TG) by the glycerol phosphate oxidase phenol 4-aminoantipyrine peroxidase method, and apolipoprotein A1 (apo-A1) and apolipoprotein B (apo B) were assayed using immunoturbidimetric assays (ABX Diagnostics, Shefford, UK). High-density lipoprotein cholesterol (HDL-C) was assayed using a second-generation homogenous direct method (Roche Diagnostics, Burgess Hill, UK). All these tests were performed on a Cobas Mira analyser (Horiba ABX Diagnostics, Nottingham, UK). The laboratory participated in the RIQAS (Randox International Quality Assessment Scheme; Randox Laboratories, Dublin, Ireland) scheme which is CRC calibrated. Low-density lipoprotein cholesterol (LDL-C) was estimated using the Friedewald formula. Non-HDL-C was estimated using the formula: non-HDL-C = (TC) − (HDL-C).

Small-dense LDL apolipoprotein B (sdLDL apoB) (density range 1.044–1.063 g/mL) was isolated from plasma adjusted to density of 1.044 g/mL and ultracentrifuged at 100,000 rpm (435,680 × *g*) for 5 h at 4°C using a Beckman Optima TLX bench top ultracentrifuge fitted with TLA 120.2 fixed angle rotor (Beckman Coulter UK) (19). ApoB in SdLDL was determined using the method described above.

### Biomarkers

An in-house, antibody sandwich ELISA technique using anti-human CRP antibodies, calibrators, and controls from Abcam (Cambridge, UK) was used to measure hsCRP. TNFα, adiponectin, leptin, resistin, MCP-1, E-selectin, P-selectin, resistin, and ICAM-1 were all measured using DuoSet ELISA development kits from R&D Systems (Abingdon, UK).

Glycated hemoglobin (HbA1c) and fasting blood glucose were measured using the standard laboratory methods in the Department of Clinical Biochemistry, Central Manchester University Hospitals National Health Service Foundation Trust. Insulin was determined in plasma using Mercodia ELISA kits from Diagenics Ltd. (Milton Keynes, UK). Homeostatic model assessment was used to assess insulin resistance (HOMA-IR) using the following formula (20):

HOMA−IR=[insulin(mU/l)×glucose(mmol/l)]/22.5.

### Statistical Analyses

Statistical analysis was performed with SPSS for Windows, Version 16.0 (Chicago, SPSS Inc.). Two way between subject ANOVA was carried out to study the interaction between independent variables (*time since surgery* and *diabetes)* and all dependent variables (e.g., weight, LDL-C, HOMA-IR, and TNFα). Univariate analysis was done to calculate effect size. R-E-G-W-Q was selected as the *post hoc* test for *time since surgery* but not *diabetes* as the latter independent variable had only two levels (i.e., presence or absence of diabetes). As sample sizes were similar, this test offered good control of type 1 error and a superior ability to detect the difference if present. Partial *eta* squared (η^2^) was calculated to establish the variance in dependent variables attributed to independent variables. η^2^ effect was graded as 0.01 = small, 0.06 = medium, and 0.14 = large. If effect of independent variables on dependent variables was significant, pairwise comparisons were reviewed to check in which period (0–6 months post surgery or 6–12 months post surgery) the main effect lay. Effect has been reported as *F* (dF, error) = *F* value, *P*-value, η^2^ (effect size) [where *F* is effect and dF is degrees of freedom]. Differences were considered as statistically significant at *P* < 0.05.

## Results

**Figure 1:**
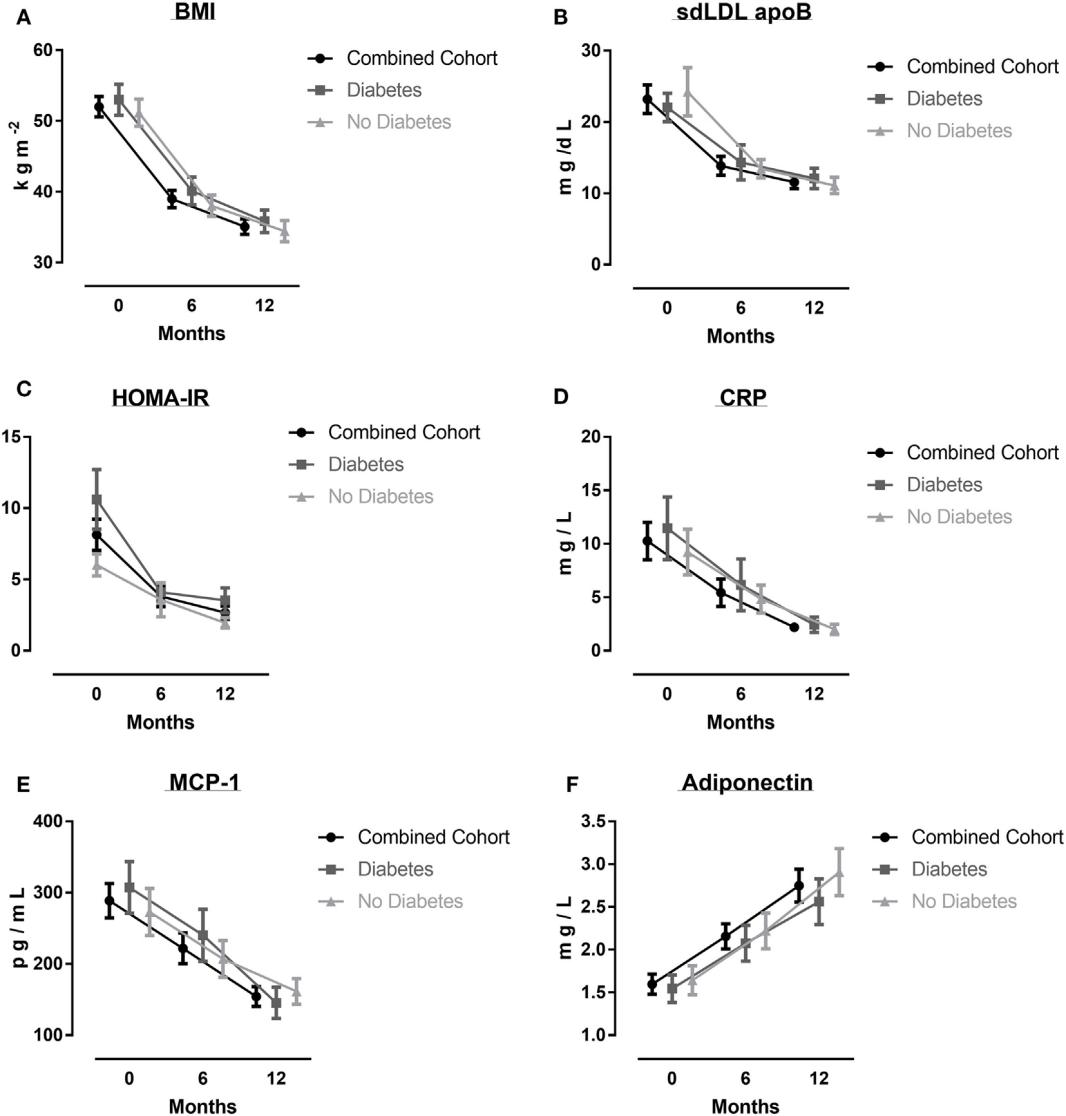
Various parameters before, and 6 and 12 months after bariatric surgery: body mass index (BMI) **(A)**, small-dense LDL apolipoprotein B (sdLDL apoB) **(B)**, HOMA-IR **(C)**, CRP **(D)**, MCP-1 **(E)**, and adiponectin **(F)**. Results are given as mean (±SEM).

Of the 37 obese patients in the study, the mean age was 49 (range, 26–63) years, mean BMI 52 (9), 17 (45%) had T2DM, 23 (60%) had hypertension, 8 (20%) were smokers, 5 (15%) were known to have ischemic heart disease, and 22 (60%) patients took a statin. In the non-diabetes patients (*n* = 20), eight (40%) were on statins (Atorvastatin equivalent dose 10–40 mg/day), whereas in the diabetes patients (*n* = 17), 15 (90%) patients were on a statin (Atorvastatin equivalent dose 10–40 mg/day). Characteristics of all patients, non-diabetes patients and diabetes patients at baseline, 6, and 12 months have been presented in Table 1 and Figure 1.

**Table 1:** Characteristics of all patients, non-diabetic group and diabetes group at baseline, 6 months, and 12 months after surgery.

	All patients (*n* = 37)			Non-diabetic patients (*n* = 20)			Diabetes patients (*n* = 17)		
	Baseline	6 months	12 months	Baseline	6 months	12 months	Baseline	6 months	12 months
Weight (kg)	140 (21)	105 (25)	93 (22)	138 (29)	105 (25)	91 (22)	143 (32)	105 (26)	95 (23)
Body mass index (kg/m^2^)	52 (9)	39 (7)	35 (7)	51 (9)	38 (7)	34 (7)	53 (9)	40 (8)	36 (7)
% EWL		46 (40–60)	63 (51–77)		46 (42–59)	69 (51–77)		49 (33–62)	60 (48–74)
Waist circumference (cm)	141 (17)	116 (15)	106 (15)	141 (16)	114 (13)	104 (15)	141 (18)	119 (18)	109 (15)
SBP (mmHg)	135 (22)	127 (20)	124 (20)	140 (25)	124 (21)	128 (23)	129 (19)	131 (19)	119 (14)
DBP (mmHg)	74 (13)	75 (13)	69 (10)	76 (15)	73 (13)	71 (11)	71 (90)	77 (12)	68 (10)
Total cholesterol (mmol/l)	4.84 (1.30)	4.94 (1.31)	4.57 (0.80)	5.26 (1.51)	5.00 (91.22)	4.76 (0.71)	4.36 (0.80)	4.87 (1.46)	4.36 (0.88)
Triglycerides (TG) (mmol/l)	1.75 (0.84)	1.45 (0.45)	1.16 (0.39)	1.55 (0.63)	1.37 (0.47)	1.12 (0.32)	1.98 (1.00)	1.54 (0.43)	1.22 (0.46)
Low density lipoprotein cholesterol (mmol/l)	2.81 (1.19)	3.04 (1.15)	2.56 (0.77)	3.28 (1.37)	3.17 (1.10)	2.75 (0.75)	2.26 (0.61)	2.89 (1.22)	2.34 (0.75)
Non-high density lipoprotein cholesterol (HDL-C, mmol/l)	3.61 (1.21)	3.66 (1.19)	3.09 (0.75)	3.99 (1.44)	3.71 (1.11)	3.25 (0.71)	3.17 (0.68)	3.59 (1.31)	2.90 (0.77)
Small-dense LDL apolipoprotein B (mg/dl)	23.19 (12.01)	13.86 (7.51)	11.57 (5.39)	24.23 (14.75)	13.46 (5.57)	11.10 (5.07)	22.04 (8.24)	14.34 (9.54)	12.10 (5.84)
ApoB (g/l)	0.96 (0.25)	1.01 (0.31)	0.88 (0.20)	1.01 (0.30)	1.01 (0.30)	0.91 (0.20)	0.90 (0.16)	1.02 (0.34)	0.84 (0.20)
HDL-C (mmol/l)	1.22 (0.29)	1.28 (0.34)	1.4 (0.34)	1.26 (0.34)	1.28 (0.37)	1.51 (0.35)	1.18 (0.22)	1.27 (0.31)	1.46 (0.34)
ApoA (g/l)	1.33 (0.23)	1.31 (0.24)	1.36 (0.22)	1.28 (0.27)	1.27 (0.25)	1.35 (0.24)	1.39 (0.17)	1.35 (0.23)	1.38 (0.21)
Adiponectin (mg/l)	1.59 (0.70)	2.15 (0.88)	2.74 (1.16)	1.64 (0.75)	2.22 (0.93)	2.90 (1.22)	1.54 (0.66)	2.07 (0.83)	2.56 (1.09)
Leptin (ng/l)	85.87 (49.50)	32.63 (26.86)	23.72 (24.06)	91.86 (51.04)	33.37 (27.45)	22.64 (15.28)	78.82 (48.19)	31.71 (26.98)	25.00 (31.98)
HOMA-IR	8.12 (6.69)	3.80 (4.27)	2.68 (2.79)	6.02 (3.48)	3.57 (5.34)	1.95 (1.63)	10.60 (8.63)	4.10 (2.50)	3.53 (3.61)
CRP (mg/l)	10.25 (10.66)	5.42 (7.69)	2.20 (2.51)	9.23 (9.52)	4.83 (5.83)	2.00 (2.08)	11.46 (12.05)	6.16 (9.70)	2.43 (2.99)
Tumor necrosis factor-alpha (pg/ml)	33.50 (39.50)	30.50 (51.09)	11.33 (29.90)	33.04 (38.36)	31.49 (40.50)	15.89 (39.17)	34.04 (41.94)	29.26 (63.32)	5.96 (11.53)
Monocyte chemoattractant protein 1 (pg/ml)	288 (147)	222 (129)	154 (84)	273 (148)	207 (116)	162 (80)	307 (149)	240 (146)	145 (91)
Intercellular adhesion molecule 1 (ng/ml)	226 (104)	184 (75)	134 (38)	224 (73)	176 (54)	135 (39)	228 (134)	193 (96)	134 (38)
E-selectin (ng/ml)	12.05 (5.80)	9.50 (3.87)	3.10 (3.67)	11.89 (5.32)	7.33 (3.11)	7.52 (3.41)	12.30 (6.48)	7.71 (4.75)	6.61 (4.00)
P-selectin (ng/ml)	36.22 (13.95)	30.37 (10.75)	23.16 (7.52)	37.13 (14.47)	29.30 (9.90)	22.87 (6.03)	35.15 (13.68)	31.70 (11.91)	23.48 (9.13)
Resistin (ng/ml)	14.78 (6.20)	14.60 (6.54)	11.30 (5.32)	14.39 (3.78)	14.66 (5.33)	12.02 (4.91)	15.23 (8.32)	14.53 (7.98)	10.44 (5.80)

*Values in mean ± SD. No patients were lost to follow-up*.

### Time since Surgery

There was a significant effect of *time since surgery* on lipoproteins, insulin resistance, mediators of systemic and vascular inflammation, and endothelial dysfunction (Table 2). A large effect size was seen on weight, BMI, waist circumference, TG, sdLDL ApoB, HOMA-IR, CRP, MCP-1, ICAM-1, E-selectin, P-selectin, leptin, and adiponectin. Medium effect size was seen on HDL-C, TNFα, and resistin and small effect size was seen on non-HDL-C. BMI and waist circumference had the largest impact of *time since surgery*. The effect of *time since surgery* was noticed mostly in the first 6 months.

**Table 2:** Shows effect size of time since surgery on dependent variables as small, medium, and large (only data with significant *P* values shown).

	*F* (dF; error)	*P*-value	Partial eta square (effect size)	*P*-value for effect at 6 months since surgery	*P*-value for effect between 6 and 12 months after surgery
Weight	31.93 (2; 70,843)	<0.0001	0.38 (large)	<0.0001	0.167
Body mass index	49.65 (2; 5,992)	<0.0001	0.49 (large)	<0.0001	0.095
Waist circumference	47.98 (2; 2,5091)	<0.0001	0.48 (large)	<0.0001	<0.05
Triglycerides (TG)	9.39 (2; 36)	<0.0001	0.15 (large)	<0.05	0.129
Non-high density lipoprotein cholesterol (HDL-C)	3.27 (2; 116)	<0.05	0.005 (small)	1.0	<0.05
Small-dense LDL apolipoprotein B	16.82 (2; 7,820)	<0.0001	0.25 (large)	<0.0001	0.86
HDL-C	6.19 (2; 11)	<0.005	0.10 (medium)	1.0	<0.05
HOMA-IR	14.32 (2; 2,319)	<0.0001	0.21 (large)	<0.0001	0.983
CRP	10.09 (2; 6,336)	<0.0001	0.16 (large)	<0.05	0.23
Tumor necrosis factor-alpha	3.19 (2; 178,782)	<0.05	0.06 (medium)	1.0	<0.05
Monocyte chemoattractant protein 1	11.14 (2; 1,599,516)	<0.0001	0.17 (large)	<0.05	<0.05
Intercellular adhesion molecule 1	12.74 (2; 632,746)	<0.0001	0.20 (large)	<0.05	<0.05
E-selectin	13.17 (2; 2,214)	<0.0001	0.20 (large)	<0.0001	1.0
P-selectin	9.34 (2; 12,327)	<0.0001	0.17 (large)	0.123	<0.05
Leptin	31.81 (2; 132,717)	<0.0001	0.38 (large)	<0.0001	0.90
Resistin	3.9 (2; 3,875)	<0.05	0.071 (medium)	1.0	<0.05
Adiponectin	13.38 (2; 92)	<0.0001	0.20 (large)	<0/05	<0.05

*Also shows P values for effect at 6 months since surgery and from 6 to 12 months after surgery*.

*F, effect that is being reported; dF, degrees of freedom*.

### Diabetes

Absence of diabetes led to a significantly greater reduction in TC, LDL-C, and non-HDL-C although the effect size was small to medium (Table 3). There was a greater reduction in TG and HOMA-IR in patients with diabetes with a small effect size (Table 3). No patients were lost to follow-up.

**Table 3:** Shows effect size of diabetes on dependent variables as small, medium, and large (only data with significant *P* values shown).

	*F* (dF, error)	*P*-value	Partial eta square (effect size)	Comments
Total cholesterol	4.71 (1, 137)	<0.05	0.043 (small)	Absence of diabetes led to greater reduction
Triglycerides	4.18 (1, 36)	<0.05	0.039 (small)	Presence of diabetes led to greater reduction
Low-density lipoprotein cholesterol	8.51 (1, 107)	<0.005	0.076 (medium)	Absence of diabetes led to greater reduction
Non–high-density lipoprotein cholesterol	4.5 (1, 116)	<0.05	0.042 (small)	Absence of diabetes led to greater reduction
HOMA-IR	6.07 (1, 2319)	<0.05	0.05 (small)	Presence of diabetes led to greater reduction

*F, effect that is being reported, dF, degrees of freedom*.

## Discussion

In this study of morbidly obese patients with or without diabetes followed over 6 and 12 months after Roux-en-Y gastric bypass surgery, we found a significant reduction in pro-atherosclerotic lipoproteins, insulin resistance, mediators of systemic and vascular inflammation, and endothelial dysfunction.

Bariatric surgery has been shown to consistently achieve significant and sustained weight loss; however, the effect on various markers of inflammation is quite variable. A recent meta-analysis evaluated the changes in blood levels of CRP and TNFα after bariatric surgery (21). There was a 54 and 81% reduction in CRP at 6 and 12 months, respectively, which is comparable to our study where we show a 47 and 78% reduction in CRP. While TNFα was only reduced by 1.3 and 1.2% at 6 and 12 months, respectively, in our study we report a 9 and 66% reduction. The much greater reduction in TNFα in our study compared with the meta-analysis could be due to a higher baseline BMI which was 52 kg/m^2^ compared to other studies included in the meta-analysis where the average BMI was <50 kg/m^2^. It could also reflect the use of Roux-en-Y surgery in our study compared to a variety of procedures, such as laparoscopic-adjustable gastric banding (LAGB), biliopancreatic diversion (BPD), or sleeve gastrectomy (SG) in the meta-analysis. There could also be significant differences in the number of patients with non-alcoholic steatohepatitis or ectopic adipose tissue, where the effect of weight loss may differ. Indeed Bachmayer et al. have reported no significant change in TNFα, MCP-1, or adiponectin, 12 months after Roux-en-Y, SG, and BPD (22). Auguet et al. have demonstrated a significant reduction in weight, insulin resistance, and CRP and circulating TNF receptors at both 6 and 12 months, with an increase in adiponectin at 6 but not 12 months after bariatric surgery (23). Sdralis et al. have demonstrated a significant fall in insulin with an increase in adiponectin with no change in TNFα even at 12 months (24). Thus, this area of research needs larger trials with a more uniform baseline weight and postoperative weight loss as well as surgical intervention such as Roux-en-Y surgery as it may lead to an exaggerated glucagon-like peptide-1 response, which may not be seen with LAGB, SG, and BPD (25).

In human studies, the role of resistin in insulin resistance and glucose metabolism is inconclusive (26, 27). Bariatric surgery results in a significant reduction in resistin levels at 12 months which correlates with insulin resistance (28). Our study confirms the significant reduction in resistin levels 12 months after bariatric surgery.

Tumor necrosis factor-alpha may play a direct role in the development of atherosclerosis through induction of ICAM-1, MCP-1, P-selectin, and E-selectin in endothelial and vascular smooth muscle cells resulting in endothelial cell apoptosis (29). Moreover, resistin augments the expression of endothelin-1, MCP-1, and ICAM-1 in endothelial cells (30, 31). The continued reduction in resistin and TNFα in the obese diabetic group up to 12 months after surgery may explain the reduction in HOMA-IR, MCP-1, and ICAM-1 in this group over the same period.

SdLDL apoB is a LDL subtype closely associated with diabetes and atherosclerosis (32), because of its greater susceptibility to undergo oxidative modification and glycation compared with more buoyant LDL (33, 34). While LAGB has been shown to modestly reduce sdLDL apoB at 12 months (35), our study shows a much more robust reduction in sdLDL apoB at 6 months with Roux-en-Y bariatric surgery.

We also demonstrate no change in apo-A1 but a significant increase in HDL-C after surgery which may indicate increased cholesterol cargo of HDL lipoproteins returning from the peripheral vasculature back to the liver, i.e., reverse cholesterol transport.

In the DM group, TC and LDL-C did not change significantly probably reflecting the fact that 90% of the DM group were already on long-term statins.

### Limitations

The 12-month follow-up of patients was relatively short. All patients recruited for this study underwent Roux-en-Y bariatric surgery; therefore, these results may not be generalized to all categories of bariatric surgery. The number of patients included in this study was limited by availability.

## Conclusion

Lipoproteins, insulin resistance, mediators of systemic and vascular inflammation, and endothelial dysfunction improve most 6 months after bariatric surgery in obese patients with and without diabetes. The greater reduction in inflammatory markers in our study compared to published meta-analyses may reflect a greater impact of Roux-en-Y surgery on weight loss achieved and may in the long-term translate to more pronounced cardiovascular benefit.

## Ethics Statement

This study was carried out in accordance with the recommendations of NRES Committee North West—Greater Manchester Central with written informed consent from all subjects. All subjects gave written informed consent in accordance with the Declaration of Helsinki. The protocol was approved by the NRES Committee North West—Greater Manchester Central.

## Author Contributions

HS, PD, and BA designed the study. RY, AS, SH, RA, JN, SA, and JH recruited patients and organized follow-ups. HS, RY, SH, YL, JS, MF, SA, TS, JH, PP, MJ, and RD did laboratory work and data analysis. RY, SH, RM, RD, PD, BA, JS, TS, RA, SD, and HS prepared the first draft. All authors contributed in revising the manuscript for important intellectual content. All authors approved of the version to be published. All authors agreed to be accountable for all aspects of the work.

## Conflict of Interest Statement

The authors declare that the research was conducted in the absence of any commercial or financial relationships that could be construed as a potential conflict of interest.
